# Distinct functions of opioid-related peptides and gastrin-releasing peptide in regulating itch and pain in the spinal cord of primates

**DOI:** 10.1038/srep11676

**Published:** 2015-06-29

**Authors:** Heeseung Lee, Mei-Chuan Ko

**Affiliations:** 1 Department of Anesthesiology & Pain Medicine, School of Medicine, Ewha Womans University, Seoul 158-710, South Korea; 2 Department of Physiology & Pharmacology, Wake Forest University, Winston-Salem, NC 27157, USA; 3 Department of Dermatology, Wake Forest University, Winston-Salem, NC 27157, USA; 4 Center for Comparative Medicine Research, School of Medicine, Wake Forest University, Winston-Salem, NC 27157, USA

## Abstract

How neuropeptides in the primate spinal cord regulate itch and pain is largely unknown. Here we elucidate the sensory functions of spinal opioid-related peptides and gastrin-releasing peptide (GRP) in awake, behaving monkeys. Following intrathecal administration, β-endorphin (10–100 nmol) and GRP (1–10 nmol) dose-dependently elicit the same degree of robust itch scratching, which can be inhibited by mu-opioid peptide (MOP) receptor and GRP receptor (BB_2_) antagonists, respectively. Unlike β-endorphin, which produces itch and attenuates inflammatory pain, GRP only elicits itch without affecting pain. In contrast, enkephalins (100–1000 nmol) and nociceptin-orphanin FQ (3–30 nmol) only inhibit pain without eliciting itch. More intriguingly, dynorphin A(1–17) (10–100 nmol) dose-dependently attenuates both β-endorphin- and GRP-elicited robust scratching without affecting pain processing. The anti-itch effects of dynorphin A can be reversed by a kappa-opioid peptide (KOP) receptor antagonist nor-binaltorphimine. These nonhuman primate behavioral models with spinal delivery of ligands advance our understanding of distinct functions of neuropeptides for modulating itch and pain. In particular, we demonstrate causal links for itch-eliciting effects by β-endorphin-MOP receptor and GRP-BB_2_ receptor systems and itch-inhibiting effects by the dynorphin A-KOP receptor system. These studies will facilitate transforming discoveries of novel ligand-receptor systems into future therapies as antipruritics and/or analgesics in humans.

Patients with chronic liver, kidney or skin diseases often suffer from changed sensory modalities such as itch/pruritus^1^,^2^,^3^. Although anticonvulsants, antidepressants, and opioid receptor antagonists seem to be helpful in some forms of chronic itch, most anti-pruritic medications are not mechanism-based^1^. Thus, there is a strong need for more research on the cause of itch, in order to advance the discovery of efficacious therapy for the treatment of itch originated centrally from diverse nervous system disorders.

Several clinical studies have revealed the antipruritic efficacy of nonselective opioid receptor antagonists in patients with cholestasis^2^,^4^, indicating an increased opioidergic tone contributing to itch in cholestatic patients. Interestingly, when plasma extracts from patients with cholestatic itch were microinjected into the medullary dorsal horn of monkeys, it elicited naloxone-reversible facial scratching activity in monkeys^5^. These early findings indicate an important role of endogenous opioid peptides in mediating itch scratching in both humans and nonhuman primates. Opioid receptor subtypes including mu-, kappa-, delta-opioid, and nociceptin/orphanin FQ peptide (N/OFQ) (i.e., MOP, KOP, DOP, and NOP) receptors mediate distinct physiological functions^6^. For example, it is known that synthetic MOP receptor agonists elicit itch and KOP receptor agonists inhibit itch^7^,^8^,^9^. However, it is not clear how diverse opioid neuropeptides contribute to different sensory modalities in primates. As dorsal horn neurons receive sensory information from primary afferents that innervate a large area of dermatomes and deeper tissues of the body^10^, it is pivotal to know the functional evidence of opioid-related neuropeptides with different binding affinities to MOP, KOP, DOP, and NOP receptors in regulating itch and pain in the spinal cord of primates.

In the past few years, there have been several exciting advances in identifying non-opioid neuropeptides, cells and neural circuits centrally in rodent models of itch^11^,^12^. One of the central itch mediators is the gastrin-releasing peptide (GRP) and its cognate receptor (BB_2_ receptor)^13^. GRP has been used to elicit scratching activity in rodents^14^. Using BB_2_ receptor mutant mice, an elegant study demonstrated that BB_2_ receptors selectively mediate itch scratching, but not pain behavior, in the spinal cord of mice^15^. Moreover, BB_2_ receptors are highly expressed in the spinal cord of monkeys displaying excessive scratching^16^ and serum GRP levels in patients with atopic dermatitis are higher than those in healthy human subjects^17^. These recent findings pinpoint the key role of the GRP-BB_2_ receptor system in mediating itch sensation. However, there is no functional study to investigate the basic characteristics such as the magnitude and duration of itch-eliciting effects of spinally delivered GRP as compared to MOP receptor-mediated itch, a well-documented phenomenon in the clinical setting^9^, and to determine how differently both BB_2_ and MOP receptors regulate itch and pain in primates.

There is now an extensive body of literature documenting that the anatomical, neurochemical, and neuropharmacological aspects of receptors are similar between nonhuman primates and humans^18^,^19^,^20^. Conversely, the translational potential of ligands and targets discovered in rodents to primates remains to be established. For example, the transient receptor potential vanilloid type1 (TRPV1)-expressing neurons are demonstrated to mediate different itch-eliciting actions in mice^21^. However, a recent clinical trial concluded that a TRPV1 antagonist SB705498 is unlikely to be of symptomatic benefit for histaminergic and non-histaminergic itch^22^. Furthermore, rodents do not display robust scratching responses when they are injected spinally with morphine, which is known to induce itch in humans^23^,^24^. In contrast, spinal administration of morphine produces analgesia and itch simultaneously in both nonhuman primates and humans^7^,^9^. Therefore, the nonhuman primate model of spinally elicited itch serves as a translational bridge which not only validates drugs for ameliorating morphine-induced itch^25^,^26^, but also identifies promising ligands which produce morphine-like analgesia without eliciting itch sensation^27^,^28^.

Given that intrathecal delivery procedure in nonhuman primates has been established to provide the sound evidence of cause and effect for diverse ligands regulating itch and pain^8^,^27^,^28^, it is important to conduct pharmacological studies in awake behaving nonhuman primates to study neuropeptides and their cognate receptors. Therefore, the aim of this study was to address a longstanding, yet unanswered, fundamental question, namely, what are the functional consequences of neuropeptides in regulating itch and pain in the spinal cord of primates? In particular, we conducted a large-scale, translational nonhuman primate study to determine (1) effects of opioid-related neuropeptides and GRP on eliciting itch scratching and inhibiting inflammatory pain, (2) selective versus general effectiveness of MOP and BB_2_ receptor antagonists as anti-itch agents, and (3) the functional role of the dynorphin A-KOP receptor system in itch and pain processing.

## Results

**Figure 1:**
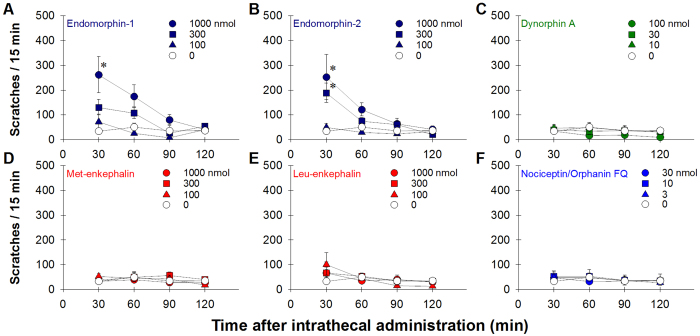
Itch scratching responses elicited by opioid-related neuropeptides delivered intrathecally in monkeys. Behavioral responses were video-recorded and quantified for each 15-min session every 30 min after intrathecal administration. Each value represents mean ± S.E.M. (n = 6). Symbols represent different dosing conditions for the same monkeys. Asterisk represents a significant difference from the vehicle condition at corresponding time point (p < 0.05).

**Figure 2:**
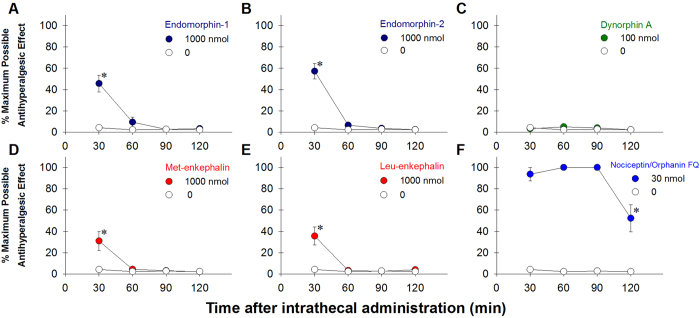
Antihyperalgesic effects produced by opioid-related neuropeptides delivered intrathecally in monkeys. The tail-withdrawal latencies were measured and expressed as % maximum possible antihyperalgesic effect against carrageenan-induced hyperalgesia in 46 °C water, every 30 min after intrathecal administration. Each value represents mean ± S.E.M. (n = 6). Symbols represent different dosing conditions for the same monkeys. Asterisk represents a significant difference from the vehicle condition from the time point 30 min to the corresponding time point (p < 0.05).

We first investigated the magnitude and duration of opioid-related neuropeptide-elicited scratching activity in the first group of monkeys (n = 6). Following lumbar intrathecal administration, both MOP receptor preferring neuropeptides, endomorphin-1 [F(3,15) = 6.3; p < 0.05] and endomorphin-2 [F(3,15) = 7.9; p < 0.05], dose-dependently elicited mild-to-moderate scratching responses, which peaked at the first observation period (i.e., ~30 min after administration) and subsided within the first hour (Fig. 1A,B). KOP (dynorphin A(1–17), [F(3,15) = 0.9; p > 0.05]), DOP (met-enkephalin, [F(3,15) = 0.5; p > 0.05] and leu-enkephalin, [F(3,15) = 0.2; p > 0.05]) and NOP (N/OFQ, [F(3,15) = 0.1; p > 0.05]) receptor preferring neuropeptides did not lead to a significant increase of scratching responses (Fig. 1C–F). These neuropeptides were examined over a wide dose range by using a single dosing procedure to detect any behaviorally active doses. It is worth noting that intrathecal dynorphin A produced long-lasting hindlimb paralysis, which was not reversible by naloxone, in rats^29^. Due to the safety reason, dynorphin A was only studied up to 100 nmol herein, and no paralysis was observed in monkeys during or after these studies. The highest dose studied for each neuropeptide was then tested in the assay of carrageenan-induced hyperalgesia. For both MOP and DOP receptor preferring neuropeptides, endomorphin-1 [F(1,5) = 17.1; p < 0.05], endomorphin-2 [F(1,5) = 40.5; p < 0.05], met-enkephalin [F(1,5) = 7.0; p < 0.05] and leu-enkephalin [F(1,5) = 10.1; p < 0.05]) at 1000 nmol produced mild-to-moderate antihyperalgesic effects (Fig. 2A,B,D,E). The KOP receptor peptide dynorphin A at 100 nmol, [F(1,5) = 0.1; p > 0.05], did not produce antihyperalgesia (Fig. 2C). In contrast, the NOP receptor neuropeptide N/OFQ at 30 nmol, [F(1,5) = 313.5; p < 0.05] produced a full antihyperalgesic effect, which continued throughout the 2-hour observation period (Fig. 2F).

**Figure 3:**
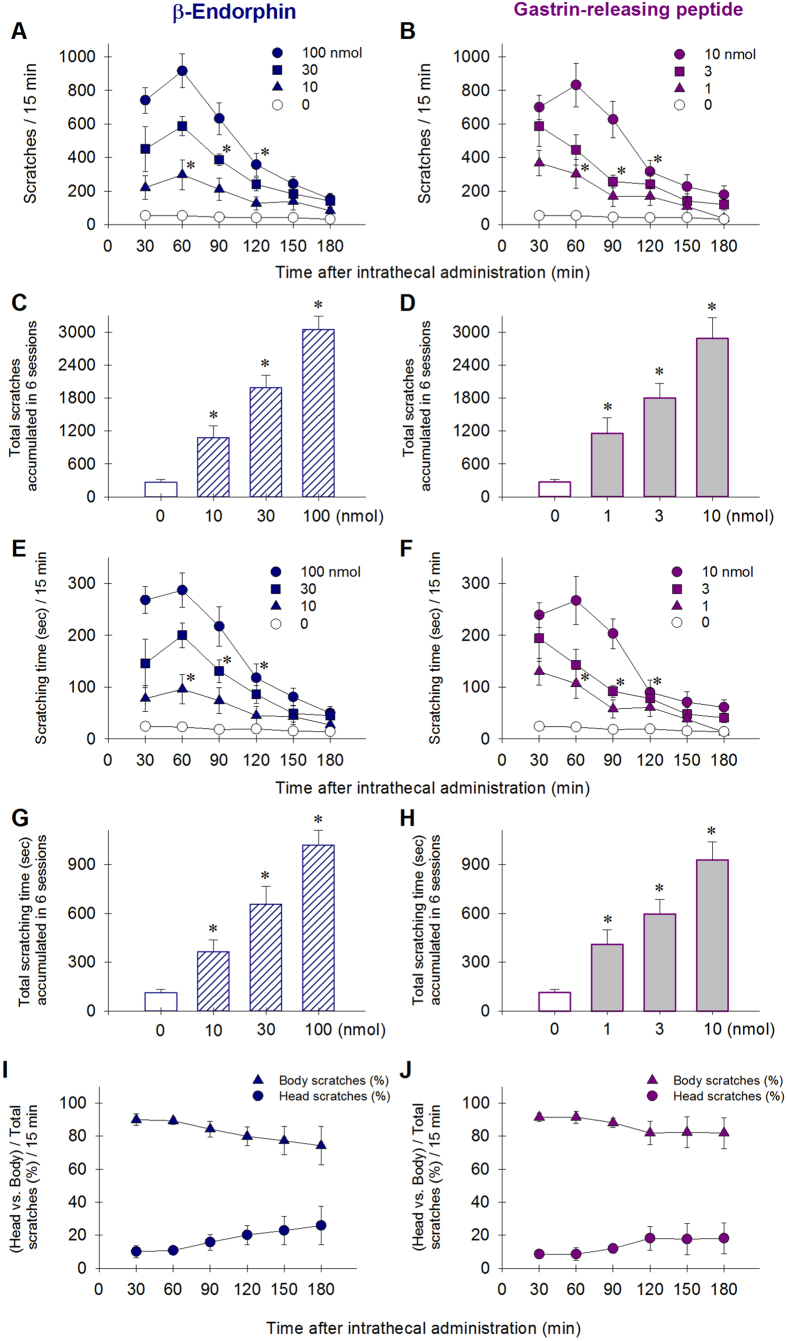
Robust scratching responses elicited by β-endorphin and GRP delivered intrathecally in monkeys. Behavioral responses were video-recorded and quantified for each 15-min session every 30 min after intrathecal administration. Each value represents mean ± S.E.M. (n = 6). Symbols represent different dosing conditions for the same monkeys. **A**,**B**: Time course of scratching responses in each 15-min session during a 3-hour observation period. **C**,**D**: the number of total scratching responses sampled throughout the entire 3-hour observation period. **E**,**F**: Time course of scratching time in each 15-min session. **G,H**: the total scratching time accumulated from 6 15-min sessions. **I**,**J**: the percentage of head versus body scratches. For panels **A,B**, **E**,**F** and **I**,**J**, asterisk represents a significant difference from the vehicle condition from the time point 30 min to the corresponding time point (p < 0.05). For panels **C**,**D** and **G**,**H**, asterisk represents a significant difference from the vehicle condition (p < 0.05).

**Figure 4:**
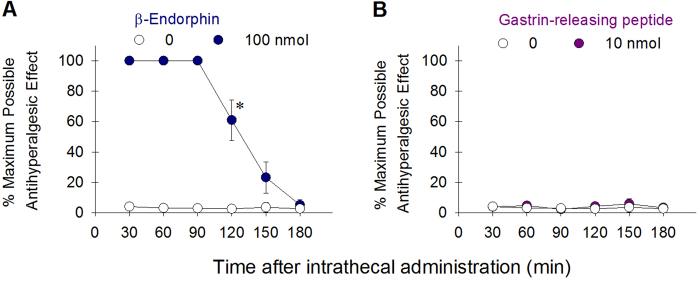
Comparison of antihyperalgesic effects produced by β-endorphin and GRP delivered intrathecally in monkeys. Each value represents mean ± S.E.M. (n = 6). Asterisk represents a significant difference from the vehicle condition from the time point 30 min to the corresponding time point (p < 0.05). See Fig. 2 for other details.

To side-by-side compare the itch-eliciting effects of β-endorphin and GRP, we determined the dose-responses for the magnitude and duration of both peptide-elicited scratching in terms of scratching responses, scratching time, and the ratios between body and head scratches in the second group of monkeys (n = 6). Following intrathecal administration, both β-endorphin [F(3,15) = 69.5; p < 0.05] and GRP [F(3,15) = 16.1; p < 0.05] dose-dependently elicited robust scratching responses, which peaked approximately at the 30-min time point, then gradually subsided throughout the 3-hour time course (Fig. 3A,B). Accumulated scratching responses from 6 observation sessions (i.e., 15 min/session) shows the dose-dependency afforded by both β-endorphin (10–100 nmol, [F(3,20) = 36.3; p < 0.05]) and GRP (1–10 nmol, [F(3,20) = 15.7; p < 0.05]) (Fig. 3C,D). Similar scratching patterns by both neuropeptides are not only revealed by the scratching numbers (Fig. 3A–D), but also by the scratching time (Fig. 3E–H). In addition, the ratios of head versus body scratching were similar between β-endorphin- and GRP-elicited scratching. Approximately 80–90% of body scratching occurred following intrathecal administration; this ratio remained unchanged for both β-endorphin [F(5,30) = 0.9; p > 0.05] and GRP [F(5,30) = 0.5; p > 0.05] (Fig. 3I,J). We further determined the potential antihyperalgesic effects of both peptides in the assay of carrageenan-induced hyperalgesia. Intrathecal β-endorphin 100 nmol significantly attenuated carrageenan-induced hyperalgesia and its antihyperalgesic effects lasted for 2 hours [F(2,10) = 265.5; p < 0.05] (Fig. 4A). In contrast, GRP 10 nmol did not produce antihyperalgesic effects (Fig. 4B).

**Figure 5:**
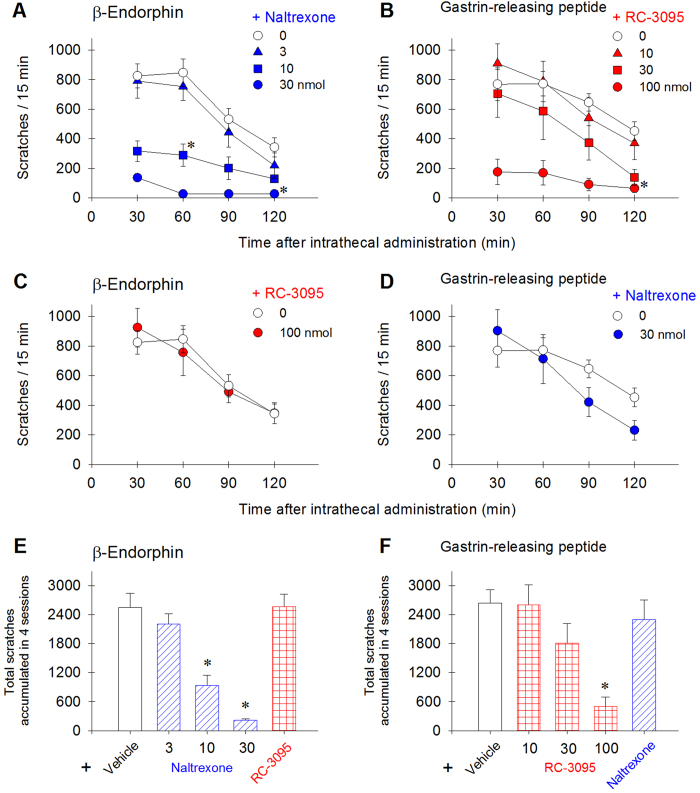
Selective inhibitory effects of MOP and BB_2_ receptor antagonists on intrathecal β-endorphin (100 nmol)- and GRP (10 nmol)-elicited scratching in monkeys. Each value represents mean ± S.E.M. (n = 6). **A**,**B**: effects of naltrexone and RC-3095 on β-endorphin- and GRP-elicited scratching, respectively. **C**,**D**: the effect of RC-3095 on β-endorphin-elicited scratching, as compared to the effect of naltrexone on GRP-elicited scratching. E-F: the number of total scratching responses sampled throughout the entire 2-hour observation period. For panels **A**,**B** and **C**,**D**, asterisk represents a significant difference from the vehicle condition from the time point 30 min to the corresponding time point (p < 0.05). For panels **E**,**F**, asterisk represents a significant difference from the vehicle condition (p < 0.05).

**Figure 6:**
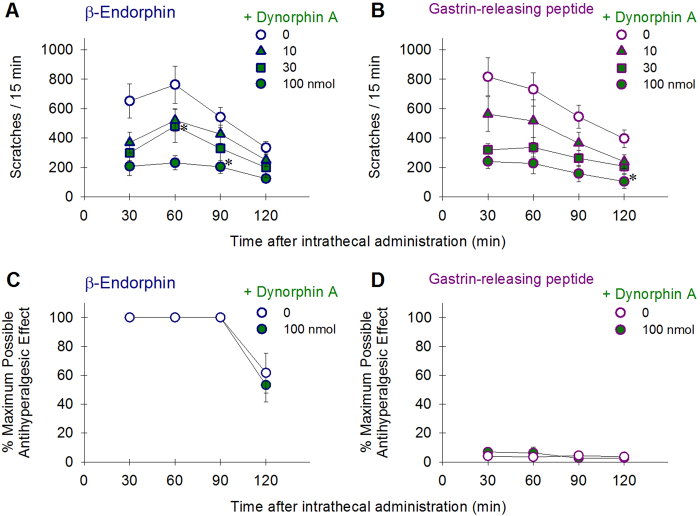
Effects of dynorphin A on intrathecal β-endorphin (100 nmol)- and GRP (10 nmol)-induced behavioral responses. Each value represents mean ± S.E.M. (n = 6). **A**,**B**: Inhibitory effects of dynorphin A on both β-endorphin- and GRP-elicited scratching responses. **C**,**D**: Lack of inhibitory effect of dynorphin A on β-endorphin-induced antihyperalgesia, as compared to no changes on the nociceptive threshold by GRP with or without dynorphin A. Asterisk represents a significant difference from the vehicle condition from the time point 30 min to the corresponding time point (p < 0.05).

To elucidate the receptor mechanisms underlying β-endorphin- and GRP-induced scratching, either the opioid receptor antagonist naltrexone (3–30 nmol, selective for MOP receptors) or the BB_2_ receptor antagonist RC-3095 (10–100 nmol) was co-administered intrathecally with neuropeptide to determine and compare their effectiveness in the third group of monkeys (n = 6). Both naltrexone ([F(4,20) = 22.9; p < 0.05]) and RC-3095 ([F(4,20) = 10.8; p < 0.05]) dose-dependently attenuated β-endorphin (100 nmol)- and GRP (10 nmol)-elicited scratching, respectively (Fig. 5A,B). However, unlike naltrexone, 100 nmol of RC-3095 failed to block β-endorphin-induced scratching (Fig. 5C,E). Naltrexone at 30 nmol was not effective in attenuating GRP-induced scratching responses (Fig. 5D,F). We further investigated whether the KOP receptor neuropeptide dynorphin A can modulate behavioral effects generated by β-endorphin or GRP. Intrathecal dynorphin A (10–100 nmol) dose-dependently attenuated robust scratching responses elicited by β-endorphin [F(3,15) = 20.3; p < 0.05] and GRP [F(3,15) = 8.0; p < 0.05] (Fig. 6A,B). However, 100 nmol of dynorphin A did not attenuate β-endorphin-induced antihyperalgesic effects [F(1,5) = 1.8; p > 0.05] (Fig. 6C). The same dose of dynorphin A, when combined with GRP, did not produce antihyperalgesia [F(1,5) = 0.3; p > 0.05] (Fig. 6D). In order to validate the involvement of spinal KOP receptors in the anti-itch effects of dynorphin A, we gave 4 subjects with intramuscular administration of a KOP receptor-selective antagonist, nor-binaltorphimine 3 mg/kg. This dosing regimen is known for producing KOP receptor antagonist effects in monkeys^8^,^30^. We found that 1-day pretreatment with nor-binaltorphimine significantly blocked the inhibitory effects of dynorphin A against β-endorphin-elicited scratching (Fig. S1).

## Discussion

Both itch and pain are unpleasant sensory experiences accompanied with different behavioral responses. By using nonhuman primate behavioral assays, this study is the first to define the functional roles of diverse opioid-related neuropeptides and GRP in regulating itch and pain in the spinal cord of primates. Four major novel findings are reported herein. First, opioid-related neuropeptides, depending on their receptor selectivity and efficacy, can differentially elicit itch scratching and ameliorate inflammatory pain. Second, both β-endorphin and GRP elicited similar magnitude and duration of robust scratching responses; unlike β-endorphin regulating both itch and pain, GRP only elicits itch sensation. Third, both spinal MOP and BB_2_ receptors can independently modulate itch. Fourth, without affecting nociceptive processing, dynorphin A attenuates β-endorphin- and GRP-induced itch by activating spinal KOP receptors.

It is known that synthetic MOP receptor agonists produce analgesia, but they elicit itch in both nonhuman primates and humans^9^,^31^. In this study, there are different degrees of itch-eliciting and pain-inhibiting effects between endomorphins and β-endorphin. The [^35^S]GTPγS binding stimulation characterized endomorphins and β-endorphin as low and high efficacy agonists at MOP receptors, respectively^32^. Both endomorphin-1 and endomorphin-2 elicited mild-to-moderate scratching responses and produced partial antihyperalgesia. In contrast, β-endorphin produced robust scratching and full antihyperalgesia. This difference illustrates a correlation between the *in vitro* ligand efficacy and the magnitude of ligand effects elicited *in vivo*^33^. Interestingly, these efficacy-dependent MOP receptor-mediated itch-eliciting effects can be observed in monkeys^25^,^34^, but not in mice^35^. For example, DAMGO, a widely used control ligand as a full MOP receptor agonist, has higher efficacy than morphine^32^. Intrathecal DAMGO elicited a greater magnitude of scratching (~1,200 scratches/15 min) than morphine (~600 scratches/15 min) in monkeys^34^. In contrast, intrathecal DAMGO and morphine both only elicited mild scratches (i.e., ~30 scratches/30 min) in mice^35^. This functional difference in itch scratching documents a species difference in the pharmacological actions of spinal MOP receptor-expressing neurons.

DOP and NOP receptor preferring neuropeptides did not significantly increase scratching responses, but they produced different degrees of antihyperalgesic effects. DOP receptor neuropeptides, met-enkephalin and leu-enkephalin, only produced transient partial antihyperalgesia. The DOP receptor mRNA level is relatively low or undetectable in the spinal cord of primates^36^,^37^, which may explain a minimal role of the enkephalin-DOP receptor system in pain processing in monkeys. In contrast, NOP receptor neuropeptide N/OFQ produced full antihyperalgesia which lasted for 1.5 hours. Based on a series of anatomical, neurobiological, and pharmacological studies, the spinal N/OFQ-NOP receptor system has been indicated to play a crucial role in pain processing of both rodents and primates^19^,^38^,^39^. Like MOP receptors, NOP receptors are coupled to Gi/Go proteins and activation of NOP receptor inhibits forskolin-stimulated cAMP production and calcium currents, activates potassium channels, and inhibits basal and stimulated release of various neurotransmitters including substance P^38^. More importantly, spinal NOP receptor agonists produce MOP receptor agonist-comparable antinociceptive effects in rodents and nonhuman primates under different pain modalities^19^,^38^,^39^, which facilitates the development of NOP receptor-related ligands as spinal analgesics without itch side effect. Co-localization of MOP and NOP receptor immunoreactivity in the superficial laminae of the rat spinal cord was not observed and both receptors were expressed predominantly on different fiber systems^40^. If such anatomical evidence can be established in the dorsal horn neurons of primates, it may explain distinct pharmacological actions of MOP and NOP receptor agonists.

Interestingly, both β-endorphin and GRP elicited robust scratching responses. Unlike MOP receptor agonist-elicited itch in rodents as mentioned above, GRP is a neuropeptide which can centrally elicit robust scratching activity in both rodents and primates^24^,^41^. The magnitude and duration of scratching elicited by both neuropeptides are similar based on their scratching number, time, and location in monkeys. To our knowledge, GRP is the first non-opioid peptide identified in the spinal cord of primates which can elicit MOP receptor agonist-comparable scratching activity. However, unlike MOP receptor agonists including β-endorphin, GRP did not produce antihyperalgesic effects. These findings together validate the translatability of somatosensory function of spinal GRP from mice^15^ to monkeys and conclude that spinal GRP selectively elicits itch with a minimum role in regulating pain in primates. Some cholestatic patients experienced itch and analgesia and their symptoms responded to opioid receptor antagonists that can ameliorate itch but cause opioid-like withdrawal discomfort^2^,^4^. Based on functional evidence of opioid-related neuropeptides in primates, β-endorphin but not endomorphins and enkephalins, could be the key neuropeptide for mediating such effects. As β-endorphin and GRP are two key neuropeptides eliciting robust scratching activity in primates, it will be important to compare both peptide levels in the cerebrospinal fluid of different populations of patients suffering from chronic itch.

Selective MOP and BB_2_ receptor antagonists, naltrexone and RC-3095, dose-dependently attenuated β-endorphin- and GRP-elicited robust scratching, respectively. However, a functionally MOP receptor-selective dose of naltrexone 30 nmol did not block GRP-induced scratching. The opposite was true for RC-3095. Effects of both antagonists provide pharmacological evidence of the functional selectivity indicating that the spinal β-endorphin-MOP receptor and GRP-BB_2_ receptor systems independently mediate itch in primates. Although intrathecal morphine-induced mild and transient scratching could be inhibited by a BB_2_ receptor antagonist^35^, the present study does not support the notion that the BB_2_ receptor is required for opioid-induced itch^35^. Both MOP and BB_2_ receptor mRNA are present in the spinal cord of primates^16^,^36^,^42^. It is important to further investigate whether there are two distinct populations of dorsal horn neurons, each expressing either MOP or BB_2_ receptors, in primates. More importantly, both MOP and BB_2_ receptors are the only two receptor systems identified so far for mediating robust itch scratching in primates and supported by well-grounded pharmacological evidence (i.e., agonists elicit itch which can be blocked by corresponding receptor antagonists)^25^,^34^. Future development of mechanism-based antipruritics may use both spinal MOP and BB_2_ receptor systems as a pharmacological basis to design bifunctional MOP-BB_2_ receptor antagonists as potential antipruritics.

Another intriguing finding is that KOP receptor neuropeptide dynorphin A(1–17) attenuated both β-endorphin- and GRP-elicited robust scratching without affecting pain processing and the anti-itch effects of dynorphin A could be reversed by a KOP receptor antagonist. Although reduced activity of the endogenous dynorphin A-KOP receptor system has been implicated for the increased scratching activity^43^,^44^, there is no direct functional evidence in terms of behavioral effects of dynorphin A on scratching responses. The present study is the first to provide the functional consequence of spinal KOP receptor activation by dynorphin A. The anti-itch effect of KOP receptor agonists was first identified in the mid-1980s^45^. There have been some rodent and nonhuman primate studies, indicating that KOP receptor agonists with different chemical structures have a broad application as antipruritics against scratching responses elicited by diverse pruritogens^8^,^30^,^43^. In particular, low doses of KOP receptor agonists are effective for inhibiting scratching and higher doses of KOP receptor agonists are required to produce antinociceptive effects which are associated with sedation^8^,^30^,^46^,^47^. These early findings have led to the development of a KOP receptor agonist, nalfurafine, which is effective in treating patients with uremic pruritus^48^. Interestingly, a recent study has identified specific inhibitory interneurons, B5-I neurons, which express dynorphin A. Acute inhibition of B5-I neurons increased scratching and KOP receptor agonists and antagonists can decrease and increase scratching, respectively in the mouse spinal cord^44^. It will be valuable to further investigate whether patients with chronic itch have a lower level of dynorphin A in the cerebrospinal fluid.

In summary, by examining both behavioral and pharmacological factors, this study elucidates the functional roles of opioid-related neuropeptides and GRP in regulating itch and pain in the spinal cord of primates. Only activating spinal MOP or BB_2_ receptors can independently elicit profound scratching activity. The potential up-regulation of the β-endorphin-MOP receptor and/or GRP-BB_2_ receptor systems and down-regulation of the dynorphin A-KOP receptor system may intertwine in patients with chronic itch. MOP and NOP receptor preferring neuropeptides may be the key mediators for inhibiting pain processing in individuals under inflammatory pain. This translational nonhuman primate behavioral model with spinal delivery of ligands bridges a scientific gap in the functional roles of neuropeptides in primates, provides physiological relevance to patients with changed sensory modalities, and facilitates transforming discoveries of novel ligand-receptor systems into future therapies in humans.

## Methods

### Subjects

Eighteen adult male and female rhesus monkeys (*Macaca mulatta*) weighing between 6.1 to 13.4 kg were used. These monkeys were individually housed. Their daily diet consisted of approximately 25 to 30 biscuits (Purina Monkey Chow; Ralston Purina Co., St. Louis, MO, USA), fresh fruit, and water *ad libitum*. All monkeys had been previously trained in the warm water tail-withdrawal assay and acclimated to being video-recorded in-cage. They were housed in facilities accredited by the Association for the Assessment and Accreditation of Laboratory Animal Care (AAALAC) International. The study protocols were approved by the Animal Care and Use Committee at the University of Michigan (Ann Arbor, MI, USA) and Wake Forest University (Winston-Salem, NC, USA). All animal care and experimental procedures were conducted in accordance with the *Guide for the Care and Use of Laboratory Animals* as adopted and promulgated by the United States National Institutes of Health (Bethesda, MD, USA).

### Procedures

#### Itch Scratching Responses

Monkeys were recorded in their home cages in order to evaluate if they displayed increasing scratching behavior, which has been demonstrated to be associated with an itch sensation^7^,^34^. The quantification of scratching is described in *SI Methods*.

#### Nociceptive Responses

The warm water tail-withdrawal latency in 46 °C water after the carrageenan administration^31^ was used to measure the antihyperalgesic effects of neuropeptides. Briefly, carrageenan (2 mg) was administered subcutaneously in the monkey’s tail to elicit hyperalgesic responses. The measurement of antihyperalgesia is described in *SI Methods*.

#### Data Analysis

Mean values (mean ± SEM) were calculated from individual values for all behavioral endpoints. Comparisons were made for the same monkeys across all test sessions in the same experiment. Individual tail-withdrawal latencies were converted to the percentage of maximum possible antihyperalgesic effects, as defined in *SI Methods*.

#### Drugs

Naltrexone HCl, opioid-related neuropeptides (National Institute on Drug Abuse, Bethesda, MD, USA), GRP, nor-binaltorphimine HCl (Tocris Bioscience, Minneapolis, MN, USA) and RC-3095 (Sigma-Aldrich, St. Louis, MO, USA) were dissolved in sterile water. The neuropeptide alone or combined with the antagonist was delivered intrathecally at a total volume of 1 mL. A detailed description of the lumbar intrathecal drug delivery has been previously described^8^,^34^. The neuropeptide was delivered intrathecally with a 10-day inter-injection interval as previous studies did^8^,^25^.

## Additional Information

**How to cite this article**: Lee, H. and Ko, M.-C. Distinct functions of opioid-related peptides and gastrin-releasing peptide in regulating itch and pain in the spinal cord of primates. *Sci. Rep.* **5**, 11676; doi: 10.1038/srep11676 (2015).

## Supplementary Material

Supplementary Information
