# Supplementary material for: Distinct functions of opioid-related peptides and gastrin-releasing peptide in regulating itch and pain in the spinal cord of primates
Source: Sci Rep. 2015 Jun 29;5:11676. doi: 10.1038/srep11676 (PMC4483774; doi:10.1038/srep11676)
Supplement: Supplementary Information [file srep11676-s1.pdf]

Supplementary Information

Distinct functions of opioid-related peptides and gastrin-releasing peptide in regulating itch and pain in the spinal cord of primates

H. Lee and M.C. Ko

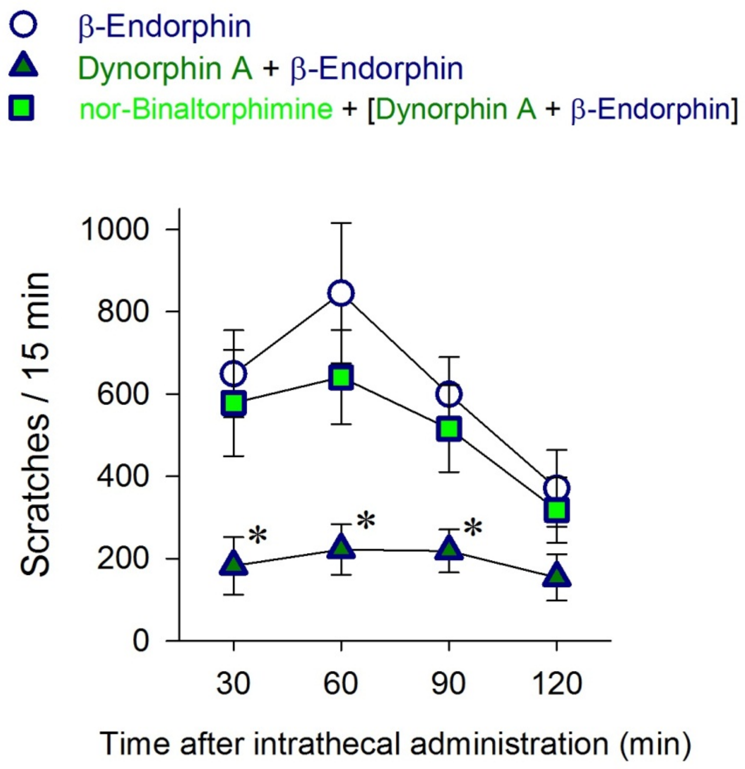

Figure S1.

Blockade of the KOP receptor antagonist nor-binaltorphimine on the inhibitory effects of dynorphin A(1-17) against intrathecal β-endorphin-elicited scratching responses in monkeys. Dynorphin A (100 nmol) was given intrathecally in combination with β-endorphin (100 nmol). Inhibitory effects of dynorphin A on β-endorphin-elicited scratching were studied 10 days before and 1 day after intramuscular administration of nor-binaltorphimine 3 mg/kg. Each value represents mean ± S.E.M. (n=4). Symbols represent different dosing conditions for the same monkeys. Asterisk represents a significant difference between conditions with or without nor-binaltorphimine treatment at corresponding time point ($p<0.05$).

SI Methods

Procedures

Itch Scratching Responses

Recording was conducted in 15-min intervals for 2-3 hours after subjects received intrathecal administration of neuropeptides, and scored by individuals who were blinded to experimental conditions. A scratch was defined as one brief scraping contact of the forepaw or hind paw on the skin surface area above the shoulder (defined as head scratches, i.e., dermatomes corresponding to cervical nerves C1-C4 and trigeminal nerves) or the area below the shoulder (defined as body scratches, i.e., skin dermatomes corresponding to cervical nerves C5-C8, all thoracic, lumbar and sacral nerves) [1]. The ratio of head versus body scratching was used to determine if β-endorphin and GRP elicited different scratching patterns. Total scratching responses and accumulated scratching time within each 15-min session were used to evaluate and compare the magnitude and duration of scratching elicited by each neuropeptide.

Nociceptive Responses

To produce inflammation-associated hyperalgesia, carrageenan (2 mg/tail in 0.1 mL of saline) was injected subcutaneously in the terminal 5 cm of the monkey's tail. This hyperalgesic response was manifested as a reduced tail-withdrawal latency from a maximum value of 20 sec to approximately 2-3 sec in 46 ^{o}C water; it peaked at 60 min and sustained for 5-6 hr after administration [2]. Each neuropeptide was delivered intrathecally approximately at the 2-hr time point after the carrageenan administration. Subjects' tail-withdrawal latencies in 46 ^{o}C water were measured 30 min before and every 30 min for 2-3 hours after intrathecal administration in order to compare and determine the potential antihyperalgesic effects of neuropeptides. All responses were measured by individuals who were blinded to experimental conditions.

Data Analysis

Individual tail-withdrawal latencies were converted to the percentage of maximum possible antihyperalgesic effects, as defined by the formula, [(test latency – latency

before drug administration) / (cutoff latency 20 sec – latency before drug administration)] x 100% [2]. The magnitude and duration of scratching behavior including the number of scratches and accumulated scratching time, and % of maximum possible antihyperalgesic effects in each test session were analyzed by two-way analysis of variance (ANOVA) with repeated measures followed by the Newman-Keuls test for multiple *post hoc* comparisons. Total scratching responses accumulated in multiple sessions were analyzed by one-way ANOVA followed by the Dunnett test for multiple comparisons. The criterion for significance for all tests was set at $p < 0.05$.

SI References

1. Ding, H., *et al.* Supraspinal actions of nociceptin/orphanin FQ, morphine and substance P in regulating pain and itch in non-human primates. *Br J Pharmacol*, DOI: 10.1111/bph.13124 (2015).
2. Sukhtankar, D.D., Lee, H., Rice, K.C. & Ko, M.C. Differential effects of opioid-related ligands and NSAIDs in nonhuman primate models of acute and inflammatory pain. *Psychopharmacology (Berl)* **231**, 1377-1387 (2014).
